# Genome-Wide Identification, Classification, and Expression Analysis of *14-3-3* Gene Family in *Populus*

**DOI:** 10.1371/journal.pone.0123225

**Published:** 2015-04-13

**Authors:** Fengxia Tian, Tan Wang, Yuli Xie, Jin Zhang, Jianjun Hu

**Affiliations:** 1 College of Life Science and Technology, Nanyang Normal University, 1638 Wolong Road, Nanyang, Henan, China; 2 State Key Laboratory of Tree Genetics and Breeding, Key Laboratory of Tree Breeding and Cultivation of the State Forestry Administration, Research Institute of Forestry, Chinese Academy of Forestry, Beijing, China; University of Delhi South Campus, INDIA

## Abstract

**Background:**

In plants, 14-3-3 proteins are encoded by a large multigene family and are involved in signaling pathways to regulate plant development and protection from stress. Although twelve *Populus* 14-3-3s were identified based on the *Populus trichocarpa* genome V1.1 in a previous study, no systematic analysis including genome organization, gene structure, duplication relationship, evolutionary analysis and expression compendium has been conducted in *Populus* based on the latest *P*. *trichocarpa* genome V3.0.

**Principal Findings:**

Here, a comprehensive analysis of *Populus 14-3-3* family is presented. Two new *14-3-3* genes were identified based on the latest *P*. *trichocarpa* genome. In *P*. *trichocarpa*, fourteen *14-3-3* genes were grouped into ε and non-ε group. Exon-intron organizations of *Populus 14-3-3s* are highly conserved within the same group. Genomic organization analysis indicated that purifying selection plays a pivotal role in the retention and maintenance of *Populus 14-3-3* family. Protein conformational analysis indicated that *Populus* 14-3-3 consists of a bundle of nine α-helices (α1-α9); the first four are essential for formation of the dimer, while α3, α5, α7, and α9 form a conserved peptide-binding groove. In addition, α1, α3, α5, α7, and α9 were evolving at a lower rate, while α2, α4, and α6 were evolving at a relatively faster rate. Microarray analyses showed that most *Populus 14-3-3s* are differentially expressed across tissues and upon exposure to various stresses.

**Conclusions:**

The gene structures and their coding protein structures of *Populus 14-3-3s* are highly conserved among group members, suggesting that members of the same group might also have conserved functions. Microarray and qRT-PCR analyses showed that most *Populus 14-3-3s* were differentially expressed in various tissues and were induced by various stresses. Our investigation provided a better understanding of the complexity of the *14-3-3* gene family in poplars.

## Introduction

Plant growing in nature constantly sense their environment and adapt to changes by using a range of biochemical and molecular mechanism [1]. And all these biological processes are controlled by signal transduction and metabolism regulation that have been known to occur via phosphorylation-mediated transition of protein states [2]. In many cases, to complete their regulatory actions, these phosphorylated proteins must physically associate with the specialized adapter proteins, which are known as 14-3-3 phosphoserine/phosphothreonine (pSer/pThr) binding protein [3–5].

*14-3-3* genes encode a ubiquitous family of highly conserved eukaryotic proteins from fungi to humans and plants with several molecular and cellular functions [6]. It was discovered in 1976 during a study of the soluble acidic proteins of the mammalian brain and was named on the basis of fraction number during DEAE-cellulose chromatography and location after starch gel electrophoresis [7]. They regulate activities of a wide array of target proteins via protein-protein interactions which involves binding with pSer/pThr residues in the target proteins [8]. Through the functional modulation of a wide range of binding partners, 14-3-3 proteins are involved in many biologically important process, including cell cycle regulation, metabolism control, apoptosis, protein trafficking, stress response, and control of gene transcription [6,9–13].

The 14-3-3 proteins can form both homo- and heterodimers [14]. Structural studies confirmed a dimeric nature of 14-3-3 proteins and revealed that each monomer consists of nine antiparallel α-helices [15,16]. A large 40 Å wide deep channel located in the center of a cup-shaped 14-3-3 protein dimer contains two amphipathic grooves [12]. All isoforms recognize two high-affinity phosphorylation-dependent 14-3-3 binding motifs: RSX**pS**XP (mode 1) and RX(F/Y)X**pS**XP (mode 2), where X is any amino acid and **pS** represents a phosphoserine [17–19]. Each monomer in the dimer is capable of interacting with a separate target protein [13]. The dimeric property of 14-3-3s allows them to serve as scaffolds by bringing two different regions of the same protein into proximity within a single complex or two different proteins together [8].

In plants, the 14-3-3 isoform was identified as part of a protein/G-box complex and therefore named GF14 (G-box Factor 14-3-3 homologs) or GRF (G-box Regulatory Factor, or General Regulatory Factor) [20]. In the last decades, a large amount of evidence has begun to accumulate for the role of 14-3-3 proteins in plant development and stress response [6]. The first *Arabidopsis* 14-3-3 isoform GRF2 (GF14omega) was discovered as a constituent of a protein/G-box complex and implicated to be involved in regulation of gene transcription [21], subsequent studies have also shown that plant 14-3-3s have a broad range of functions [6,22,23]. To date, an increasing body of work is beginning to clarify the roles of 14-3-3s in stress response pathways in plants. There are two main ways by which environmental inputs may affect 14-3-3 activity or be affected by 14-3-3s. Firstly, environmental stimuli may activate signaling pathways that causes the phosphorylation of client proteins to which 14-3-3s then recognize and bind. Secondly, these stimuli may affect 14-3-3s themselves by means of transcriptional regulation of specific 14-3-3s or by affecting the levels of signaling molecules such as divalent cations or AMP to which 14-3-3s can bind. The post-translational modification of specific 14-3-3 isoforms may also be affected [23]. A direct evidence that 14-3-3s play a major functional role in environmental stress responses comes from the overexpression of *Arabidopsis* 14-3-3λ in cotton enhanced drought tolerance in transgenic plants [24]. 14-3-3s may also exert their effects by interacting with components of hormone signaling pathways [25].

Recently the whole genome sequencing of plants has assisted for a survey of plant 14-3-3 proteins and possible implications for their role in plant growth and developmental processes [8]. Because of the economic importance in pulp and biofuel production, the studies on the genus *Populus* have been hotspots for many years [26]. The completion of *Populus trichocarpa* genome sequence in 2006 makes it as a model tree for other tree species [27]. Despite that much is learnt about 14-3-3s in non-woody plants, the diversity of this group of proteins in woody plants is not yet known. To determine the structure and function of *14-3-3* in the *Populus* genus, we performed detailed systematic analyses of genome organization, gene structure, protein structure, and expression compendium. In this study, we report the comprehensive genomic identification and phylogenetic analysis of all fourteen members of the *14-3-3* gene family in *Populus*, as well as their expression profiles in different tissues and their responses under various abiotic stresses. Our preliminary results may provide insights to further investigate the functions of these genes.

## Materials and Methods

### Characteristics of *Populus 14-3-3* genes and phylogenetic analysis

To identify potential members of the *Populus* 14-3-3 gene family, we performed multiple database searches. Published *Arabidopsis* and rice 14-3-3 protein sequences were retrieved and used as queries in tBLASTn searches against the *P*. *trichocarpa* genome database (http://www.phytozome.net/poplar.php, release 3.0). WoLF PSORT (http://wolfpsort.org) was used to predict protein subcellular localization [28]. The pI and molecular weight were estimated using the Compute pI/Mw tool from ExPASy (http://web.expasy.org/compute_pi).

Sequences of *Medicago trucatula*, *Sorghum bicolor*, *Brachypodium distachyon*, *Vitis vinifera*, *Glycine max*, and *Physcomitrella patens* were obtained from Phytozome (http://phytozome.jgi.doe.gov/pz/portal.html) by tBLASTn searches using *Arabidopsis* and rice 14-3-3 protein sequences as queries. Multiple Sequences Alignment (MSA) of 14-3-3 protein sequences from *P*. *trichocarpa* and other eight plant species were performed using the Clustal X program (V2.1) [29]. A maximum likelihood (ML) phylogenetic tree was constructed using PhyML (V3.0) with 1,000 bootstrap replicates [30].

### Gene structure, chromosomal location and synteny analysis

The exon and intron structures were illustrated using Gene Structure Display Server (GSDS, http://gsds.cbi.pku.edu.cn) [31] by aligning the cDNA sequences with the corresponding genomic DNA sequences from Phytozome (http://www.phytozome.net/). The chromosomal locations of the *14-3-3* genes were determined using the *P*. *trichocarpa* genome browser (http://www.phytozome.net/poplar). Tandem duplicated genes were defined as adjacent homologous *14-3-3* genes on the *P*. *trichocarpa* chromosomes, with no more than one intervening gene. For synteny analysis, synteny blocks within the *P*. *trichocarpa* genome and between *P*. *trichocarpa* and *Arabidopsis thaliana* genomes were downloaded from the Plant Genome Duplication Database (PGDD, http://chibba.agtec.uga.edu/duplication/) [32] and those containing *Populus 14-3-3* genes were identified. The chromosomal locations of *14-3-3* genes were drawn using Circos software [33].

### Calculation of *K*a/*K*s values

Amino acid sequences from segmentally duplicated pairs were aligned first by Clustal X program (V2.1) [29] and the aligned sequences were subsequently transferred into original cDNA sequences using the PAL2NAL program (http://www.bork.embl.de/pal2nal/) [34], which uses the CODEML program of PAML [35] to estimate synonymous (*K*s) and nonsynonymous (*K*a) substitution rates. Divergence time (T) was calculated using a synonymous mutation rate of λ substitutions per synonymous site per year as T = *K*s/2λ (λ = 9.161029 for *Populus*) [36].

### Protein modeling, molecular conservation and structural analysis

To better understand the molecular mechanism of *Populus* 14-3-3 protein, the deduced *Populus* 14-3-3 protein sequences were modelled using the top 10 PDB closed templates structures by I-Tasser [37]. The predicted organic binding site was based on the identification of analogs with similar binding sites taking into account their BS-scores, TM-scores (a scale for measuring the structural similarity between two structures), IDEN (percentage sequence identity in the structurally aligned region), the coverage of the alignment by TM-align, the COV of the model, and the structural alignment (which is equal to the number of structurally aligned residues divided by their length). A BS-score value of >0.5 signifies a binding site prediction with high confidence. The ligand(s) in the analog structure were then transferred onto the model and the fitness of the ligand-model complex (BS-score) was calculated by comparing the local structure and sequence similarity in the binding site region.

### EST profiling and publicly available microarray data analyses

The expression profiles for each gene was obtained by evaluating its EST representation among 18 cDNA libraries derived from different tissues and/or developmental stages available at PopGenIE (http://www.popgenie.org/) [38].

The microarray data for various tissues and developmental stages available at NCBI Gene Expression Omnibus (GEO) database (http://www.ncibi.nlm.nih.gow/geo/) under the series accession numbers GSE13990 (from *P*. *balsamifera*), GSE30507 (from *P*. *maximowiczii × nigra*), and GSE13043 (from *P*. *trichocarpa*) were used for the tissue-specific expression analysis. The series GSE13990 includes Affymetrix microarray data from nine different tissue samples representing three biological replicates [39], series GSE30507 includes Affymetrix microarray data from six tissue samples representing two biological replicates [40], whereas series GSE13043 contains NimbleGen microarray data from five stem internodes (IN) from the apical bud to the base of the shoot (IN2 to IN5, IN9) in two biological replicates [41].

For abiotic and hormonal treatments, Affymetrix microarray data available in the NCBI GEO database under the series accession numbers GSE13109 (hypoxia), GSE26199 (heat) and GSE16786 were analyzed [42,43]. GSE16786 is composed of the following five subsets: GSE14893 (nitrogen limitation, genotype 1979), GSE14515 (nitrogen limitation, genotype 3200), GSE16783 (1 week after leaf wounding), GSE16785 (90 h after leaf wounding), and GSE16773 (methyl jasmonate-elicited suspension cell cultures). Probe sets corresponding to *Populus* 14-3-3 genes were identified using the online Probe Match tool POParray (http://aspendb.uga.edu/poparray). For genes with more than one probe sets, the median of expression values was considered. The expression data were gene-wise normalized. The data was normalized by the Gene Chip Robust Multiarray Analysis (GCRMA) algorithm followed by log transformation and average calculation. After normalization and log transformation of data for all the *Populus* genes present on the chip, the log signal intensity values for *Populus* probe set IDs corresponding to *14-3-3* genes were extracted for further analyses.

### Plant material, RNA isolation and real-time qRT-PCR

Plant materials were collected from clonally propagated 1-year-old hybrid poplar (*P*. *alba* × *P*.*glandulosa*) clone (84K) grown in a growth chamber under long-day conditions (16 h light/8 h dark) at 23–25°C. Six vegetative tissues (SAM-shoot apical meristem, YL-young leaf, ML-mature leaf, PS-primary stem, SS-secondary stem, and R-root) were collected from 84K. Samples were frozen immediately in liquid nitrogen, and stored at -80°C for further analysis. Three biological replicates were performed.

Total RNA was extracted using the RNeasy Plant Mini Kit (Qiagen) with on-column treatment with RNase-free DNase I (Qiagen) to remove any contamination of genomic DNA. First-strand cDNA synthesis was carried out with approximately 1 μg RNA using the SuperScript III reverse transcription kit (Invitrogen) according to the manufacturer’s procedure. Primers with melting temperatures of 58–62°C and amplicon lengths of 150–250 bp were designed using Primer3 software (http://frodo.wi.mit.edu/primer3/input.htm). qRT-PCR was conducted on LightCycler 480 Detection System (Roche, Penzberg, Germany) using SYBR Premix Taq Kit (TaKaRa, Dalian, China) according to the manufacturer’s instructions. Relative expression was calculated by the 2^-ΔΔCt^ method [44]. The *PtActin* and *PtTubulin* were used as internal controls.

## Results and Discussion

### Identification of *14-3-3* gene family in *Populus* and other plant species

To identify *14-3-3* genes in *Populus*, we performed a tBLASTn search against *P*. *trichocarpa* genome release 3.0 (http://www.phytozome.net/poplar/) using 14-3-3 protein sequences in *Arabidopsis* and rice as queries and the resulting sequences were used as secondary queries [2,45]. After manual reannotation and confirmation of the motif, total of fourteen putative *14-3-3* genes were identified. In previous study, a total of twelve *14-3-3* genes were identified in *Populus* by a genome-wide bioinformatics survey based on the *P*. *trichocarpa* genome V1.1 [46]. In this study, we further revealed two additional 14-3-3 genes in *Populus* and extended the total member to fourteen. We designated *Populus 14-3-3* genes as *PtGRF* following the nomenclature proposed in the previous study [45]. The *14-3-3* genes identified in *P*. *trichocarpa* encode proteins ranging from 238 to 306 amino acids (aa) in length, with predicted isoelectric points (pIs) ranging from 4.7 to 5.9. The detailed information of *14-3-3* genes in *Populus* were listed in Table 1.

**Table 1:** *Populus 14-3-3* gene family.

Group	Gene Name	Gene Locus	CDS (bp)	ORF (aa)	pI	MW (kDa)	PSORT predictions*
non-*ε* group	*PtGRF1/2/4a*	Potri.002G099800	789	262	4.7	29.38	nucl_plas: 5.5, plas: 5.0, nucl: 4.0, chlo: 2.0, cyto: 2.0
	*PtGRF1/2/4b*	Potri.005G162400	786	261	4.7	29.30	plas: 5.0, nucl_plas: 5.0, chlo: 4.0, nucl: 3.0, cyto: 2.0
	*PtGRF3/5/7a*	Potri.004G101700	813	270	5.2	30.47	cyto: 7.0, nucl: 3.0, chlo: 2.0, plas: 2.0
	*PtGRF3/5/7b*	Potri.017G113300	792	263	4.8	29.57	nucl_plas: 5.0, nucl: 4.0, cyto: 4.0, plas: 4.0, chlo: 2.0
	*PtGRF3/5/7c*	Potri.T147900	759	252	4.9	28.47	nucl_plas: 5.5, nucl: 5.0, cyto: 4.0, plas: 4.0
	*PtGRF6/8a*	Potri.002G103800	768	255	4.7	28.60	plas: 5.0, nucl_plas: 5.0, chlo: 4.0, nucl: 3.0, mito: 1.0
	*PtGRF6/8b*	Potri.005G157700	783	260	4.9	29.10	chlo: 5.0, plas: 4.0, nucl_plas: 4.0, nucl: 2.0, cyto: 2.0
*ε* group	*PtGRF9a*	Potri.001G392200	834	277	5	31.58	chlo: 6.0, nucl_plas: 4.0, nucl: 3.0, plas: 3.0, cysk: 2.0
	*PtGRF9b*	Potri.011G110900	786	261	4.9	29.51	nucl_plas: 5.5, nucl: 5.0, plas: 4.0, chlo: 3.0, cyto: 1.0
	*PtGRF11a*	Potri.002G097500	762	253	4.9	28.97	plas: 5.0, nucl_plas: 5.0, nucl: 3.0, cyto: 3.0, mito: 1.0, extr: 1.0
	*PtGRF11b*	Potri.005G164500	921	306	5.1	34.76	cyto: 4.0, chlo: 3.0, plas: 2.0, nucl: 1.0, mito: 1.0, extr: 1.0, cysk: 1.0
	*PtGRF12a*	Potri.008G095000	789	262	4.8	29.63	nucl: 7.0, nucl_plas: 6.5, plas: 4.0, cyto: 2.0
	*PtGRF12b*	Potri.010G159300	789	262	4.8	29.63	nucl_plas: 5.0, nucl: 4.0, plas: 4.0, cyto: 3.0, chlo: 1.0, mito: 1.0
	*PtGRF13*	Potri.001G125600	717	238	5.9	27.59	nucl: 7.0, cyto: 5.0, mito: 1.0

Gene loci are obtained from the Phytozome website (http://www.phytozome.net).

*PSORT predictions: plas (plasma membrane), cyto (cytosol), chlo (chloroplast), cysk (cytoskeleton), nucl (nuclear), mito (mitochondrion), extr (extracellular). The numbers indicate the number of nearest neighbors to the query which localize to each site.

**Fig 1:**
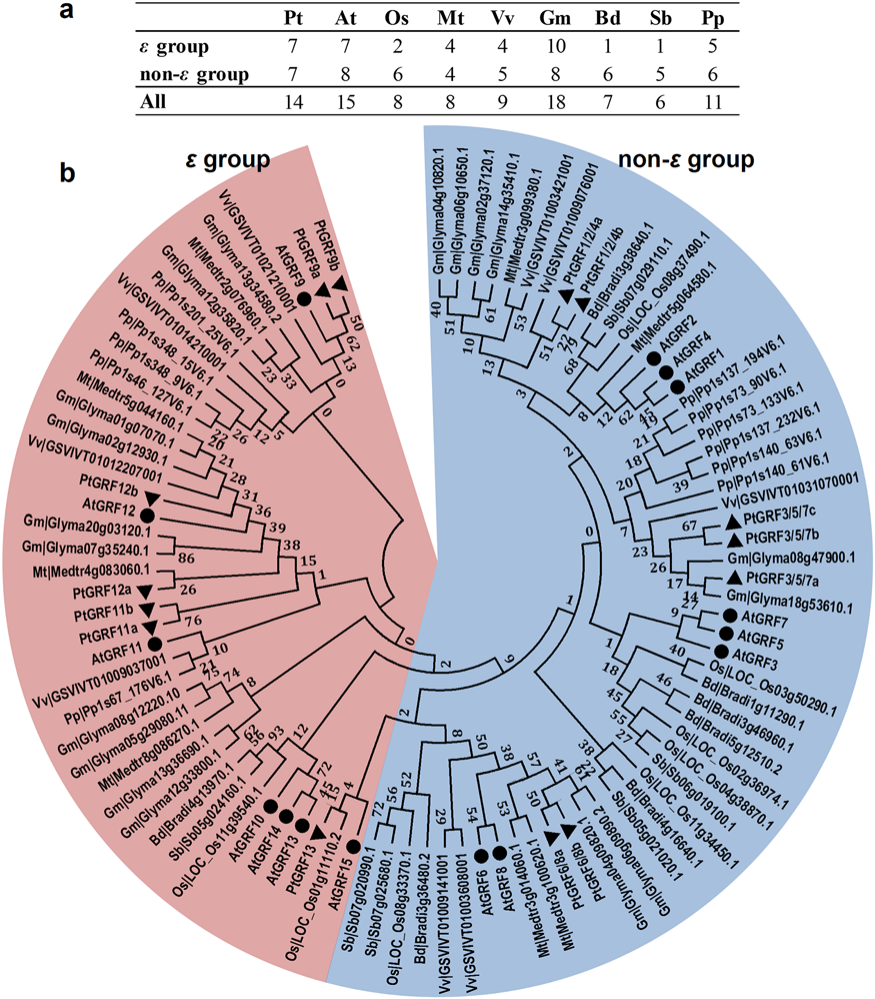
*14-3-3* family members (a) and their phylogenetic relationships (b) from nine plant species. **a.** *14-3-3* family members of *A*. *thaliana* (At), *P*. *trichocarpa* (Pt), *O*. *sativa* (Os), *M*. *truncatula* (Mt), *S*. *bicolor* (Sb), *B*. *distachyon* (Bd), *V*. *vinifera* (Vv), *G*. *max* (Gm), and *P*. *patens* (Pp). **b.** Multiple alignment of 14-3-3 proteins from nine plant species was performed using Clustal X2.1. Phylogenetic tree was constructed using full-length protein sequences by the maximum likelihood (ML) method with 1,000 bootstrap replicates. Bootstrap support values are shown on each node. The two major groups are marked with different background colors. Detail information of *14-3-3s* from nine plant species were listed in S1 Table.

**Fig 2:**
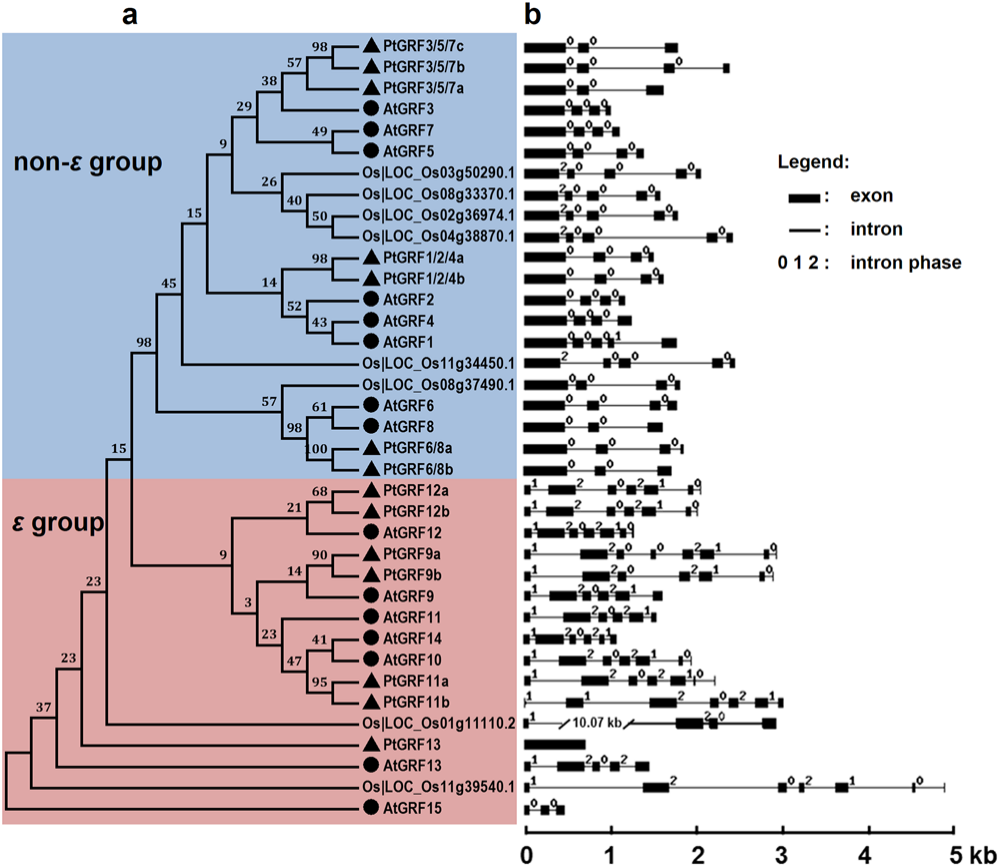
Phylogenetic analysis (a) and exon-intron structures (b) of *P*. *trichocarpa*, *Arabidopsis*, and rice *14-3-3s*. **a.** A multiple alignment of full-length 14-3-3 protein sequences from three species was executed using Clustal X2.1 and a phylogenetic tree was constructed by the maximum likelihood (ML) method with 1,000 bootstrap replicates. Bootstrap support values are shown on each node. The two major groups are marked with different background colors. **b.** Exon/intron structures of the *14-3-3* genes. Black boxes represent exons and lines represent introns. The numbers indicate the splicing phases of the *14-3-3s*: 0, phase 0; 1, phase 1; and 2, phase 2.

In order to gain insight into the evolutionary relationships among plant 14-3-3 proteins, we identified *14-3-3* genes from eight other plant species with whole genome sequences available, including moss (*Physcomitrella patens*), the monocotyledonous angiosperms *Oryza sativa*, *Sorghum bicolor* and *Brachypodium distachyon*, and the dicotyledonous angiosperms *Arabidopsis thaliana*, *Medicago truncatula*, *Glycine max*, and *Vitis vinifera* (Fig 1). All angiosperm genomes as well as the genome of the moss contained genes encoding 14-3-3 proteins. A complete list of all *14-3-3* genes identified in this study was provided in S1 Table. As comparative genomic study revealed a ratio of 1.4~1.6 putative poplar homologs for each *Arabidopsis* gene [27], it was hypothesized that *14-3-3* genes in *Populus* would be a large multi-gene family as fifteen *14-3-3s* were identified in *Arabidopsis* [45]. However, according to this study, the number of *Populus 14-3-3* genes was even smaller than that of *Arabidopsis* (Fig 1b and Table 1). It can be hypothesized that fourteen *14-3-3* genes would be sufficient for *Populus* to mediate signal transduction and a subset of *14-3-3* genes might have lost during the evolutionary process due to the functional redundancy. We named the *Populus* 14-3-3s based on their phylogenetic relationships with the *Arabidopsis* 14-3-3s. For example, PtGRF3/5/7a, PtGRF3/5/7b, and PtGRF3/5/7c were clustered in the same clade with AtGRF3, AtGRF5, and AtGRF7 (Fig 2a).

### Phylogenetic and gene structure analysis of *Populus 14-3-3* gene family

To examine the evolutionary relationships of 14-3-3 proteins from different organisms, we constructed a phylogenetic tree by maximum likelihood (ML) method using the full-length 14-3-3 protein sequence alignments of nine plant species (*P*. *trichocarpa*, *A*. *thaliana*, *O*. *sativa*, *M*. *truncatula*, *V*. *vinifera*, *G*. *max*, *B*. *distachyon*, *S*. *bicolor*, and *P*. *patens*, Fig 1). The 14-3-3 proteins of all nine plant species were classified into two major groups (*ε* group and non-*ε* group, Fig 1a), the same as described previously [47]. Noticeably, the ratios of *ε* group members to total *14-3-3* genes in monocotyledon plants (25% in *O*. *sativa*, 14.3% in *B*. *distachyon*, and 16.7% in *S*. *bicolor*) were relatively less than that in moss (45.5% in *P*. *patens*) and in dicotyledon plants (50% in *P*. *trichocarpa*, 46.7% in *A*. *thaliana*, 50% in *M*. *truncatula*, 44.4% in *V*. *vinifera*, and 55.6% in *G*. *max*) (Fig 1a). This implying that some *ε* group *14-3-3* genes in monocotyledon plants might be lost during the evolution. The retained one or two *ε* group *14-3-3* genes might play the conserved function in monocotyledon plants.

**Fig 3:**
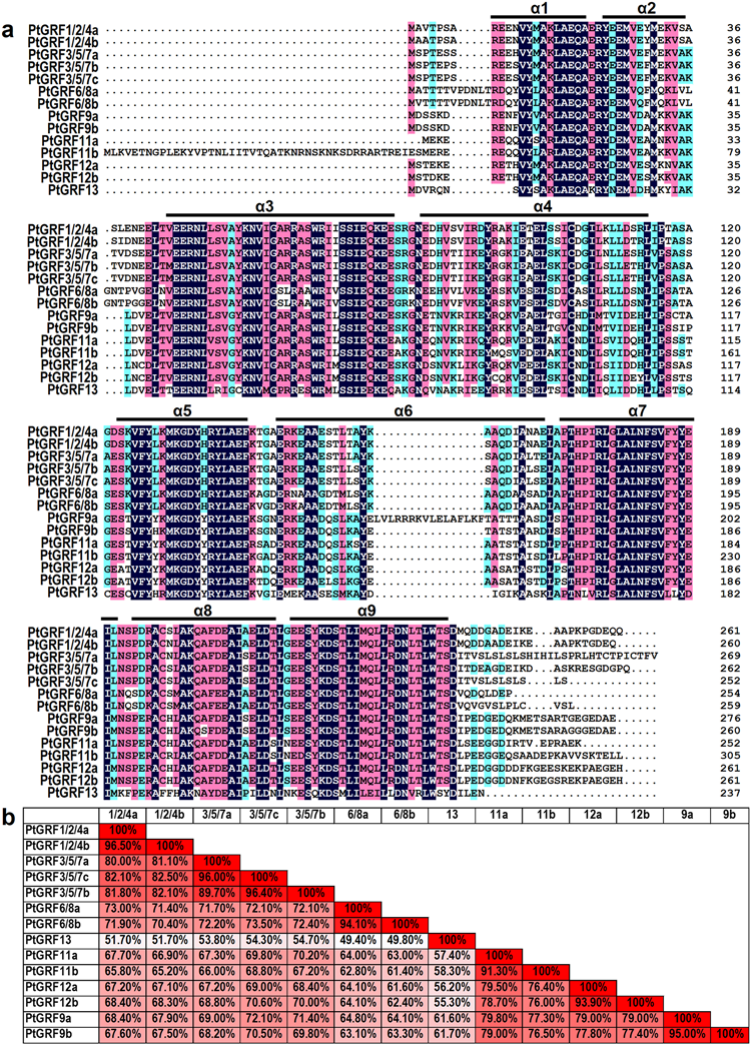
Sequence alignment and identity of *Populus* 14-3-3 proteins. **a.** Amino acid sequence alignment of the fourteen *Populus* 14-3-3 proteins. Nine α-helices were marked as α1-α9. **b.** Amino acid identity among *Populus* 14-3-3 proteins was analyzed in pairwise fashion.

To gain further insights into the structural diversity of *Populus 14-3-3* genes, we then analyzed the exon-intron organization in the coding sequence of the *P*. *trichocarpa*, *Arabidopsis*, and rice *14-3-3* genes (Fig 2). Exon-intron structural divergence within families plays a pivotal role in the evolution of multiple gene families. Generally, the positions of some spliceosomal introns are conserved in orthologous genes. In many cases, conservation of exon-intron organization in paralogous genes is high and sufficient to reveal the evolutionary relationship between introns [48]. As shown in Fig 2b, most members in each group have the similar number of exons and also exhibited nearly identical exon lengths. The intron phases are remarkably well conserved among family members, while the intron arrangements and intron phases are distinct between subfamilies (Fig 2b). An alignment of deduced *Populus* 14-3-3 proteins is shown in Fig 3a. The amino acid sequences are highly conserved except in the C-terminal and N-terminal regions. The sequence conservation among *Populus* 14-3-3 proteins was also supported by the percentage identity at amino acid level (49.4 ~ 96.5%, Fig 3b).

### Chromosomal location and duplication analysis of *Populus 14-3-3* genes

**Fig 4:**
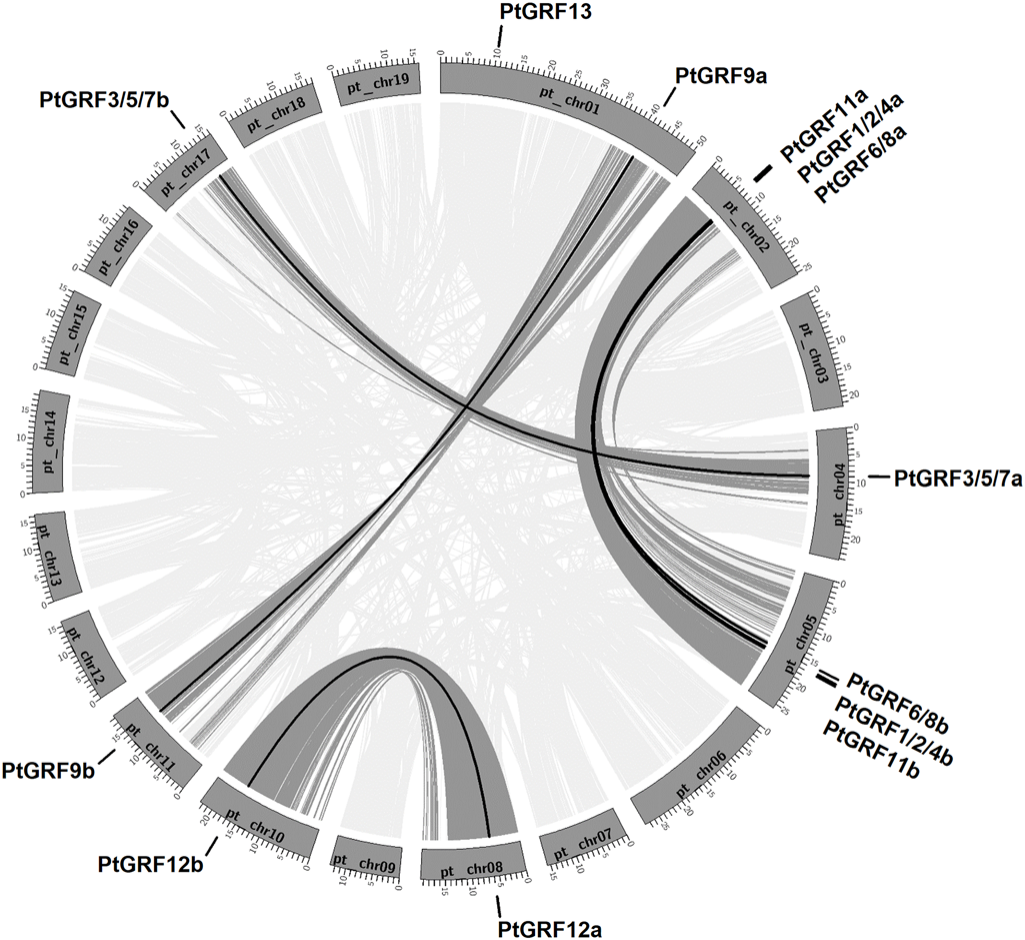
Distribution and synteny of *14-3-3*s on *Populus* chromosomes. Nineteen *Populus* chromosomes (chr01-chr19) are depicted as grey bars. Chromosome numbers are indicated in centers of each bar. *Populus 14-3-3s* are indicated by vertical black lines outer the circles. Duplicate pairs formed by whole or segmental genome duplication are connected by bold black lines.

In silico mapping of the gene locus showed that thirteen of fourteen *Populus 14-3-3* genes were mapped to eight of nineteen chromosomes (chr) unevenly, with only one gene (*PtGRF3/5/7c*) remained on as yet unmapped scaffolds (Fig 4). Previous analysis indicated that *Populus* genome has undergone at least three rounds of genome wide duplications followed by multiple segmental duplication, tandem duplication, and transposition events [49]. To determine the possible evolutionary relationship between *Populus* 14-3-3 genes and potential segmental duplications, *Populus 14-3-3* genes were mapped to the nine duplicated blocks established in the previous study [27]. The distributions of *14-3-3* genes relative to the corresponding duplicated blocks were illustrated in Fig 4. Within the identified duplicated blocks associated with the recent salicoid duplication event, twelve of fourteen (85.7%) *Populus 14-3-3* genes were preferentially retained duplicates that located in both duplicated regions. In contrasts, *PtGRF13* was located on one block and lacked duplicates on their corresponding blocks. The results indicated that dynamic rearrangement may have occurred following the segmental duplication which results in the loss of some genes.

Based on the genomic organization of *Populus 14-3-3* genes, we found that all of the six *Populus 14-3-3* paralogous gene pairs were generated by segmental duplications (Fig 4 and Table 2). *PtGRF1/2/4a* - *PtGRF1/2/4b*, *PtGRF6/8a* - *PtGRF6/8b*, and *PtGRF11a* - *PtGRF11b* were three of the paralogous gene pairs identified in this study. Interestingly, *PtGRF1/2/4a*, *PtGRF6/8a*, and *PtGRF11a* together with *PtGRF1/2/4b*, *PtGRF6/8b*, and *PtGRF11b*, were found within the same duplication blocks with identities higher than 60% (Fig 4 and Fig 3b). This arrangement, along with the closely related location in the phylogenetic tree, suggested that the three paralogous gene pairs might originate from a common ancestor which firstly underwent tandem duplication prior to the segmental duplication. Moreover, the results indicated that *Populus 14-3-3* genes had been preferentially retained at a relatively high rate of 85.7%, which was much higher than the average rate (33%) following the salicoid genome wide duplication in the *Populus* lineage [27]. This also corroborated previous reports showing that genes involved in transcription regulations and signal transductions were preferentially retained following duplications [50–53].

**Table 2:** Divergence between paralogous *14-3-3* gene pairs in *Populus*.

No.	Gene 1	Gene 2	Duplication	Ka	Ks	Ka/Ks	Date (million years ago)
1	*PtGRF1/2/4a*	*PtGRF1/2/4b*	W	0.015	0.281	0.053	15.46
2	*PtGRF3/5/7a*	*PtGRF3/5/7b*	W	0.081	0.373	0.217	20.51
3	*PtGRF6/8a*	*PtGRF6/8b*	W	0.034	0.202	0.167	11.08
4	*PtGRF9a*	*PtGRF9b*	W	0.022	0.280	0.079	15.40
5	*PtGRF11a*	*PtGRF11b*	W	0.042	0.201	0.209	11.07
6	*PtGRF12a*	*PtGRF12b*	W	0.031	0.162	0.191	8.92

To verify whether Darwinian positive selection was involved in *Populus 14-3-3* genes divergence after duplication, the substitution rate ratio of nonsynonymous (*K*a) versus synonymous (*K*s) were calculated for *Populus 14-3-3* gene pairs. Generally, *K*a/*K*s = 1 means neutral selection, *K*a/*K*s>1 means accelerated evolution with positive selection, and *K*a/*K*s<1 means purifying selection [54]. As the results showed that the *K*a/*K*s ratios of total six *Populus 14-3-3* gene pairs were less than 0.3 (Table 2), we could conclude the *Populus 14-3-3* gene family had undergone great purifying selection pressure with limited functional divergence after segmental duplications. Based on the divergence rate of 9.1×10^-9^ synonymous mutations per synonymous site year proposed for *Populus* [55], duplications of the paralogous gene pairs were estimated to occur between 8.92 and 20.51 million years ago (Table 2).

### Evolutionary relationship of *14-3-3* family between *Populus* and *Arabidopsis*

**Fig 5:**
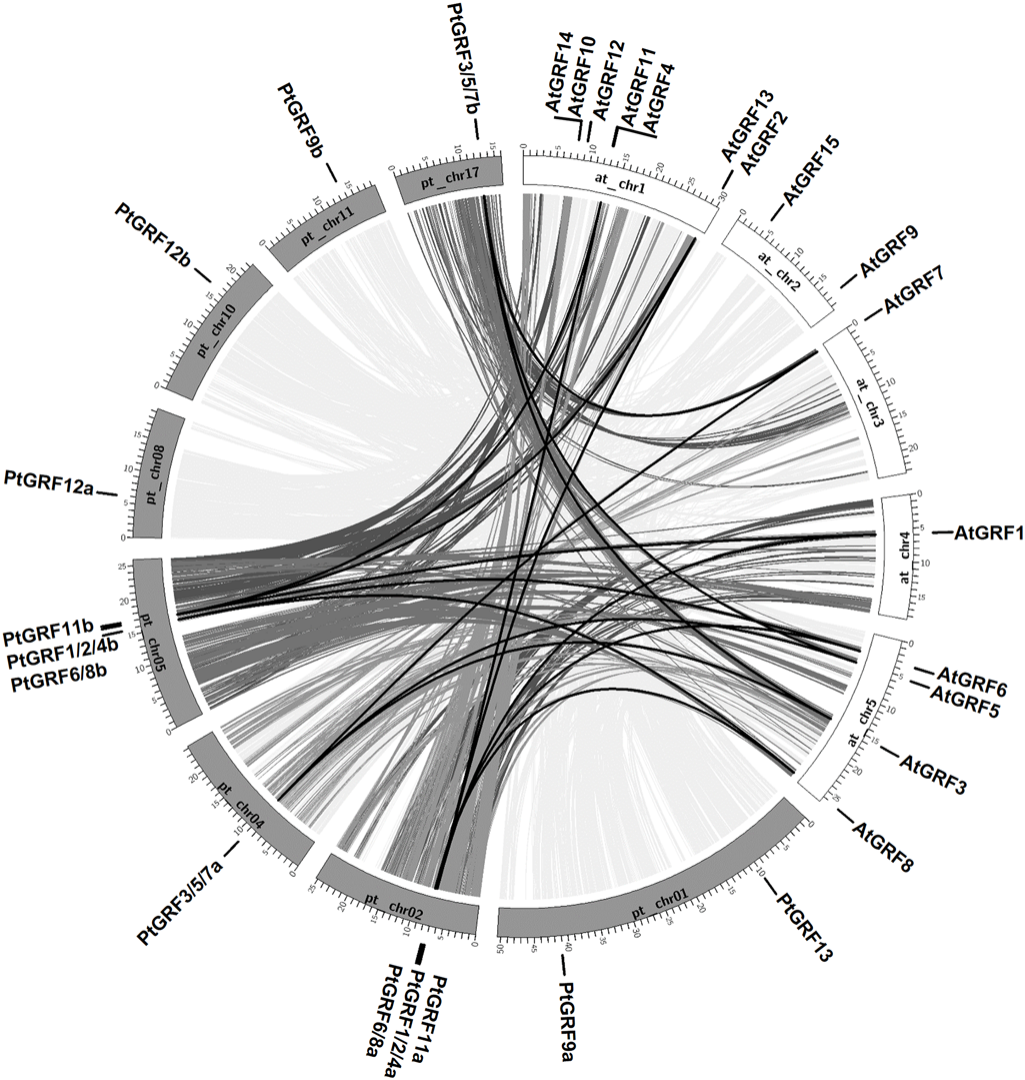
Synteny analysis of *14-3-3* genes between *Populus* and *Arabidopsis*. *Populus* and *Arabidopsis* chromosomes are depicted as grey and blank boxes, respectively. *14-3-3s* are indicated by vertical black lines outer the circles. Syntenic relationship are connected by lines between *Populus* and *Arabidopsis* chromosomes.

Comparing the sequences of all genes between genomes from different taxa provide the possibly to reconstruct the evolutionary history of each gene in its entirety [56]. To further investigate the origin and evolutionary process of *Populus 14-3-3* genes, we analyzed the comparative synteny map between *P*. *trichocarpa* and *Arabidopsis* genomes (Fig 5). Since *Arabidopsis* is one of the most important model plant species and the functions of some *Arabidopsis 14-3-3* genes have been well characterized [24,57–59]. Thus, through comparative genomics analysis we might infer the functions of *Populus 14-3-3* genes based on their *Arabidopsis* homologous.

There are one *Populus 14-3-3* gene pair was syntenic with corresponding one *Arabidopsis 14-3-3* gene (*PtGRF11a*-*PtGRF11b* corresponding to *AtGRF11*), and this pair was duplicated within the recent large-scale genome duplication event (13 Ma) in *Populus* (Table 2). More challenging for syntenic interpretation are cases where duplicated *Populus* genes corresponded to two (*PtGRF6/8a*-*PtGRF6/8b* corresponding to *AtGRF6* and *AtGRF8*) or three (*PtGRF1/2/4a*-*PtGRF1/2/4b* corresponding to *AtGRF1*, *AtGRF2*, and *AtGRF4*; *PtGRF3/5/7a*-*PtGRF3/5/7b* corresponding to *AtGRF3*, *AtGRF5*, and *AtGRF7*) *Arabidopsis* genes (Fig 5). While there are still two pairs (*PtGRF9a*-*PtGRF9b* and *PtGRF12a*-*PtGRF12b*) could not be mapped into any synteny blocks. It might because the genomes have undergone multiple rounds of significant chromosomal rearrangement, fusions, and following selective gene loss after speciation of *Populus* and *Arabidopsis* [27].

### Structural characterization of *Populus* 14-3-3 proteins

**Fig 6:**
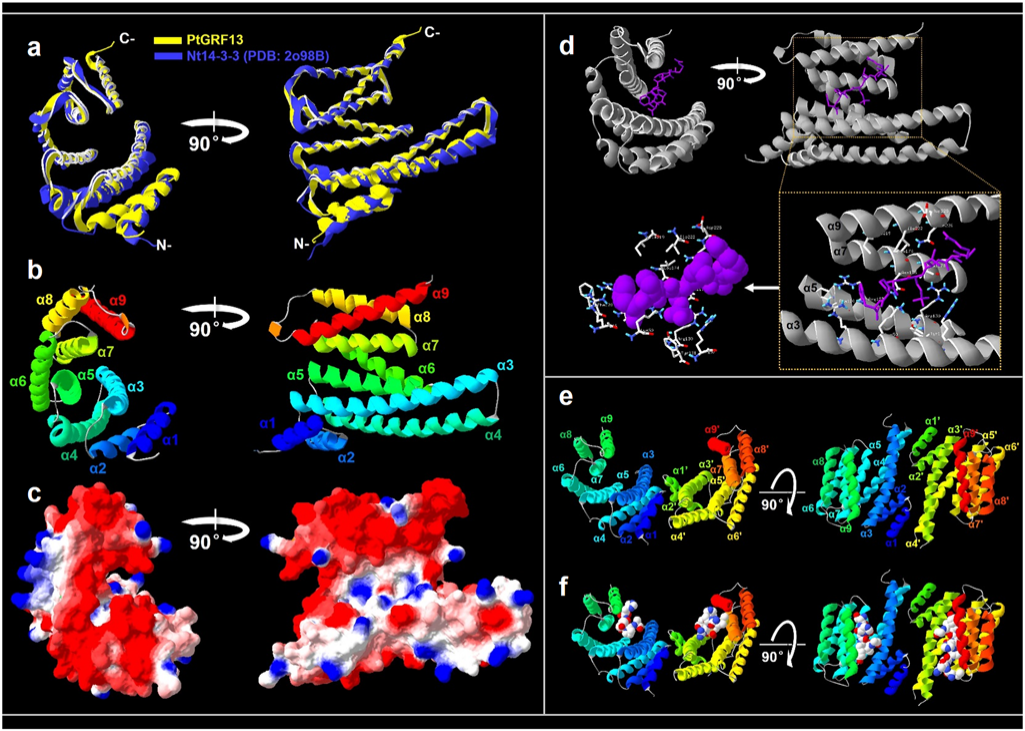
Structural analysis of *Populus* 14-3-3 protein. **a.** Structural comparison of PtGRF13 (yellow) and Nt14-3-3(PDB: 2o98B) (blue). **b.** Monomeric structure of *Populus* 14-3-3 protein. The helices are labelled α1-α9. **c.** Electrostatic surface representation of the *Populus* 14-3-3 protein. The protein surface is colored according to its electrostatic potential: blue for positive and red for negative charge. **d.** Predicted binding model of *Populus* 14-3-3 monomer and other molecule. **e.** Structure of *Populus* 14-3-3 dimer. The helices are labelled α1-α9. **f.** Predicted binding model of *Populus* 14-3-3 dimer and other molecules. Each structure is rotated 90° to show different view side of the protein.

Because of the important role of 14-3-3 in plant development and environmental stress response, some reported crystal structures of 14-3-3 proteins from different plant species have been deposited in the Protein Data Bank (PDB) database (http://www.rcsb.org/pdb/home/home.do) up to date [60,61]. In order to understand the functional mechanism of *Populus* 14-3-3 proteins, we analyzed in detail the conformational features of *Populus* 14-3-3s using computational biology. We obtained the best predicted model of totally fourteen *Populus* 14-3-3 proteins based on the ten best structural templates and the crystal structures of 14-3-3 proteins from different organisms deposited in the Protein Database (Fig 6 and S1 Fig). The quality of the modeled protein was estimated by the C-score values generated by I-TASSER software, which reflects the coverage parameters in the structural simulations and the sequence alignment with the template [37]. C-score is a confidence scoring function to assessing the quality of a prediction and estimate the accuracy of the I-TASSER software predictions, which is based on the quality of the threading alignments and the convergence of I-TASSER’s structural assembly refinement simulations. Typically, a good predicted model is obtained when the estimated level of confidence (C-score) is between -5 and 2. The level of confidence for all our predicted *Populus* 14-3-3 models were in the range of -1.57 to 1.62 (Table 3), indicating that the protein structures were constructed with high accuracy. Other parameters like TM-score and root mean square deviation (RMSD) were used to check the topology and structural similarity of the models [62].

**Table 3:** Structural-dependent modeling parameters for the *Populus* 14-3-3 protein family.

Gene Name	Gene ID	C-score	TM-score	RMSD (Å)	Template (higher Z-score)	PSI-BLAST % Identity with the template	Norm. Z-score
*PtGRF1/2/4a*	Potri.002G099800	0.31	0.75±0.10	5.2±3.4	2o98B	91	2.73
*PtGRF1/2/4b*	Potri.005G162400	0.26	0.75±0.10	5.3±3.4	2o98B	91	2.73
*PtGRF3/5/7a*	Potri.004G101700	0.01	0.71±0.11	5.9±3.7	2o98B	88	2.69
*PtGRF3/5/7b*	Potri.017G113300	0.04	0.72±0.11	5.8±3.6	2o98B	86	2.74
*PtGRF3/5/7c*	Potri.T147900	0.46	0.77±0.10	4.9±3.2	2o98B	86	2.81
*PtGRF6/8a*	Potri.002G103800	1.21	0.88±0.07	3.4±2.4	2o98B	75	2.73
*PtGRF6/8b*	Potri.005G157700	0.48	0.78±0.10	4.9±3.2	2o98B	75	2.69
*PtGRF9a*	Potri.001G392200	0.18	0.74±0.11	5.6±3.6	2o98B	72	2.47
*PtGRF9b*	Potri.011G110900	0.32	0.76±0.10	5.2±3.3	2o98B	72	2.70
*PtGRF11a*	Potri.002G097500	0.77	0.82±0.09	4.2±2.8	2o98B	71	2.76
*PtGRF11b*	Potri.005G164500	-1.57	0.52±0.15	9.8±4.6	2o98B	71	2.36
*PtGRF12a*	Potri.008G095000	0.37	0.76±0.10	5.1±3.3	2o98B	73	2.72
*PtGRF12b*	Potri.010G159300	0.5	0.78±0.10	4.9±3.2	2o98B	74	2.70
*PtGRF13*	Potri.001G125600	1.62	0.94±0.05	2.5±1.9	2o98B	52	2.80

The determined 14-3-3 crystal structures were all homo- or heterodimers. Each monomer consists of a bundle of nine α-helices (α1-α9) organized into groups of two, two, two, and three helices (Fig 3 and Fig 6). The first four are essential for formation of the dimer, which has a sizeable aperture at the subunit interface (Fig 6e). Helices α3, α5, α7, and α9 form a conserved peptide-binding groove, which has a positively charged patch on one side and a hydrophobic patch on the other (Fig 6d and 6f). We also constructed the separate phylogenetic trees based on each of α-helices. We found that the domain of helices α1, α3, α5, α7, and α9 were evolving at a low rate, while α2, α4, and α6 were evolving at a relatively fast rate (S2 Fig). These results implying that the conserved α1, α3, α5, α7, and α9 domains might play conserved functions during the evolution. The predicted *Populus* 14-3-3 structures were similar to the 14-3-3 proteins in other species [13]. Although 14-3-3 proteins commonly form dimers, the monomers are sometimes functional, depending on the target protein, 14-3-3 proteins may use the dimerization process to control their cellular activities [13]. Generally, the 14-3-3 proteins bind to their binding partners mostly in a phosphorylation-dependent manner by recognizing specific pSer/pThr containing motifs [17]. In the binding event, two processes likely occur: binding of the phosphorylated peptide to the conserved groove (α3, α5, α7, and α9; primary interaction) and interaction of the globular domain of the protein with the remaining sections of the 14-3-3 (secondary interaction) [13]. The phosphorylation and other posttranslational modifications of 14-3-3 isoforms can efficiently modulate their interaction and dimerization [63]. In addition, 14-3-3 proteins not only bind a large and diverse set of phosphoproteins but also seem to coordinate their targets in different ways [64].

### *Populus 14-3-3* genes were differentially expressed across different tissues and involved in abiotic stress responses

**Fig 7:**
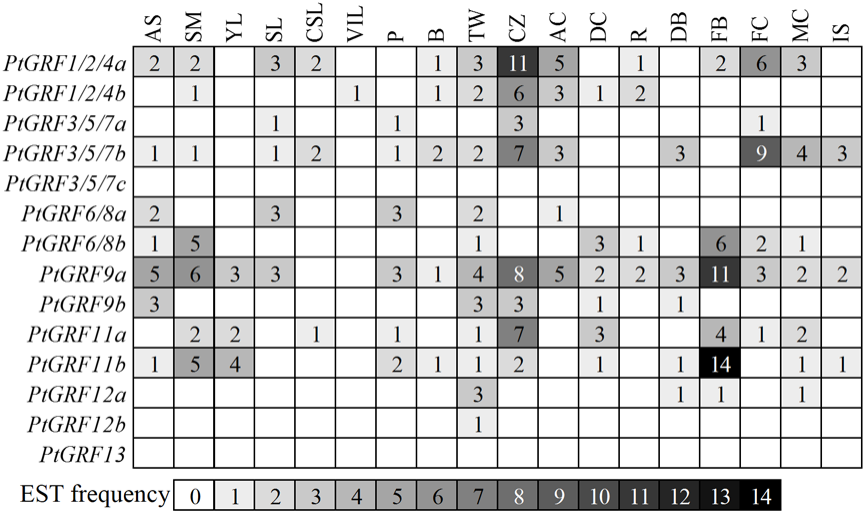
In silico EST analysis of *Populus 14-3-3* genes. EST frequency for each gene was calculated by evaluating its EST representation among 18 cDNA libraries available at PopGenIE (http://www.popgenie.org/) [38]. Color bar at bottom represents the frequencies of EST counts. AS: apical shoot, SM: shoot meristem, YL: young leaves, SL: senescing leaves, CSL: cold stressed leaves, VIL: Virus/fungus-infected leaves, P: petioles, B: bark, TW: tension wood, CZ: cambial zone, AC: active cambium, DC: dormant cambium, R: roots, DB: dormant buds, FB: flower buds, FC: female catkins, MC: male catkins, IS: imbibed seeds

Publicly available Expression Sequence Tags (ESTs) by Digital Northern and whole genome microarray provide the useful tool to survey gene expression profiles in *Populus* [65,66]. We firstly conducted a preliminary analysis of *Populus 14-3-3s* expression across different tissues and under various growth conditions by counting the frequencies of ESTs in different poplar cDNA libraries obtained from PopGenIE (http://www.popgenie.org/) (Fig 7). Most of the *Populus 14-3-3s* had a broad expression pattern across different tissues. Noticeable, *PtGRF1/2/4a* with high abundance in cambial zone while *PtGRF9a* and *PtGRF11b* with high abundance in floral buds. A study in lily has suggested 14-3-3s may play a role in the germination and elongation of pollen [67]. It was suggested that the mechanisms by which 14-3-3s act in pollen grain development may include regulation of ion channels, osmoregulation, transport of proteins from the ER and Golgi to the plasma membrane, and also in mitochondrial energy generation for tube growth [68].

**Fig 8:**
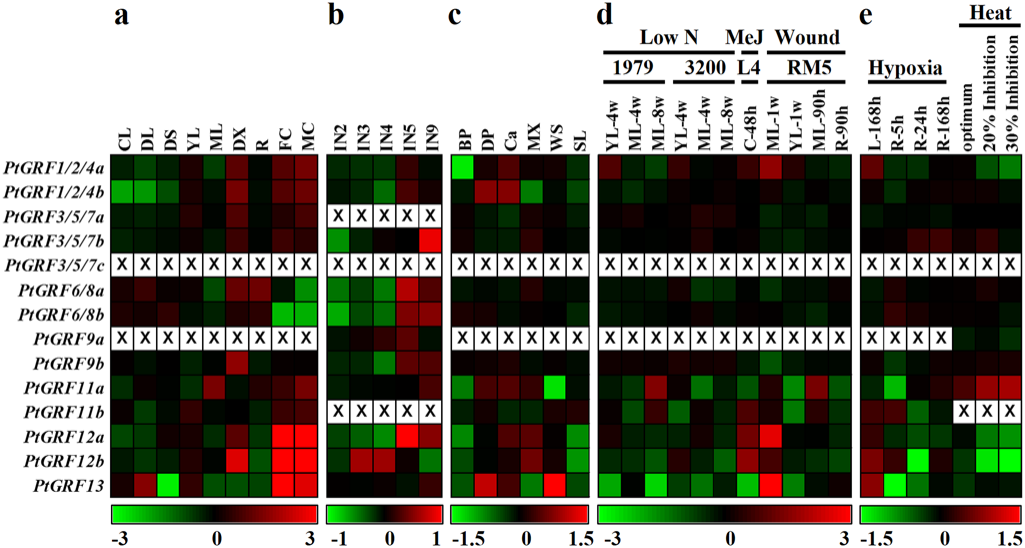
Expression profiles of *Populus 14-3-3* genes across different tissues and various abiotic stresses in different *Populus* species. **a.** Expression profiles of *Populus 14-3-3* genes across various tissues in *P*. *balsamifera*. The Affymetrix microarray data were obtained from NCBI Gene Expression Omnibus (GEO) database under the series accession number GSE13990. CL, continuous light-grown seedling; DL, etiolated dark-grown seedling transferred to light for 3 h; DS, dark-grown seedlings; YL, young leaf; ML, mature leaf; DX, differentiating xylem; R, root; FC, female catkins; MC, male catkins. **b.** Expression profiles of *Populus 14-3-3* genes at different stem development stages in *P*. *trichocarpa*. The NimbleGen microarray data were obtained from GSE17230. IN2-IN5 and IN9, stem internodes 2 to 5 and internodes 9. **c.** Expression profiles of *Populus 14-3-3* genes in different tissues of actively growing young poplar (*P*. *maximowiczii × nigra*) stems (one year old). The Affymetrix microarray data were obtained from GSE30507. BP, bark and mature phloem; DP, developing phloem; Ca, cambial zone; MX, mature xylem; WS, whole stem; SL, shoot apical meristem and leaf primordial. **d.** Expression profiles of *Populus 14-3-3* genes across various stresses and genotypes analyzed. Microarray data was obtained from GSE16786. Genotypes analyzed included: *Populus fremontii* × *angustifolia* clones 1979, 3200, and RM5, *Populus tremuloides* clones 271 and L4, and *Populus deltoids* clones Soligo and Carpaccio. Tissues analyzed included: YL, young leaves; ML, mature leaves; R, root tips; C, suspension cell cultures. Stress treatments included: low N, nitrogen limitation; MeJ, Methyl Jasmonate elicitation; Wound, sampled either one week or 90 hours after wounding. **e.** Expression profiles of *Populus 14-3-3* genes under hypoxia and heat stresses. Microarray data were obtained from GSE13109 (hypoxia) and GSE26199 (heat). Stress treatments included: Hypoxia, the leaves and root system of grey poplar (*Populus* × *canescens*) were flooded for up to 168 h; Heat, fully expanded leaf samples of *P*. *trichocarpa* were harvested at 4 physiological states as determined from prior gas exchange measurements (growth temperature: 22°C—baseline, 31.75°C—photosynthetic optimum, 38.4°C—20% inhibition of optimum and 40.5°C—30% inhibition of optimum). Color scale represents log2 expression values, green represents low level and red indicates high level of transcript abundances. Probe sets of *Populus 14-3-3s* were listed in S2 Table.

To gain more insights into the expression of *Populus 14-3-3s* in different tissues, a comprehensive analysis was conducted based on two Affymetrix (GSE13990, GSE30507) and a Nimblegen (GSE13043) microarray data [39–41]. Although these microarray datasets were performed in different platforms, they largely represented the *Populus 14-3-3s* presenting in this study (Fig 8a–8c). Five *Populus 14-3-3s* (*PtGRF1/2/4a*, *1/2/4b*, *6/8a*, *9b*, and *12b*) showed high transcript abundance in the differentiating xylem. Three *Populus 14-3-3s* (*PtGRF12a*, *12b*, and *13*) were preferentially expressed in male and female catkins (Fig 8a). During stem development, *PtGRF3/5/7b* had high accumulation in the basal stem undergoing secondary growth (internode 9), while *PtGRF12b* showed high expression level in the upper stem (internode 3 and 4) (Fig 8b). In addition, *PtGRF1/2/4b* and *13* were highly expressed in developing phloem (Fig 8c). The tissue specific expression implied that members of *Populus 14-3-3s* involved in special developmental processes. It has been proposed that *14-3-3s* may allow the growth and development of cells to be co-ordinated with the metabolic status of the plants [69]. The expression of all six cotton *14-3-3* isoforms were increased during the elongation phases of fiber development. These included the regulation of hormone signaling pathways, regulation of Myb transcription factors and alterations in activity of the H^+^-ATPase activity leading to pH changes and subsequently structural changes of the cell wall [70].

We then compared the expression patterns between *Arabidopsis 14-3-3s* and *Populus 14-3-3s* in various tissues. The *PtGRF6/8a* and *PtGRF6/8b* were syntenic with corresponding *AtGRF6* and *AtGRF8* (Fig 5), and they all had low abundance in floral organs in *Populus* and *Arabidopsis* separately (Fig 8a and S3 Fig). Although *PtGRF12a* and *PtGRF12b* could not mapped into *Arabidopsis* synteny blocks, they were highly expressed in floral organs as their orthologous AtGRF12 (Fig 8a and S3 Fig). The similar expression patterns of these orthologous between *Populus* and *Arabidopsis* implying that they might keep some conserved functions during the evolution.

**Fig 9:**
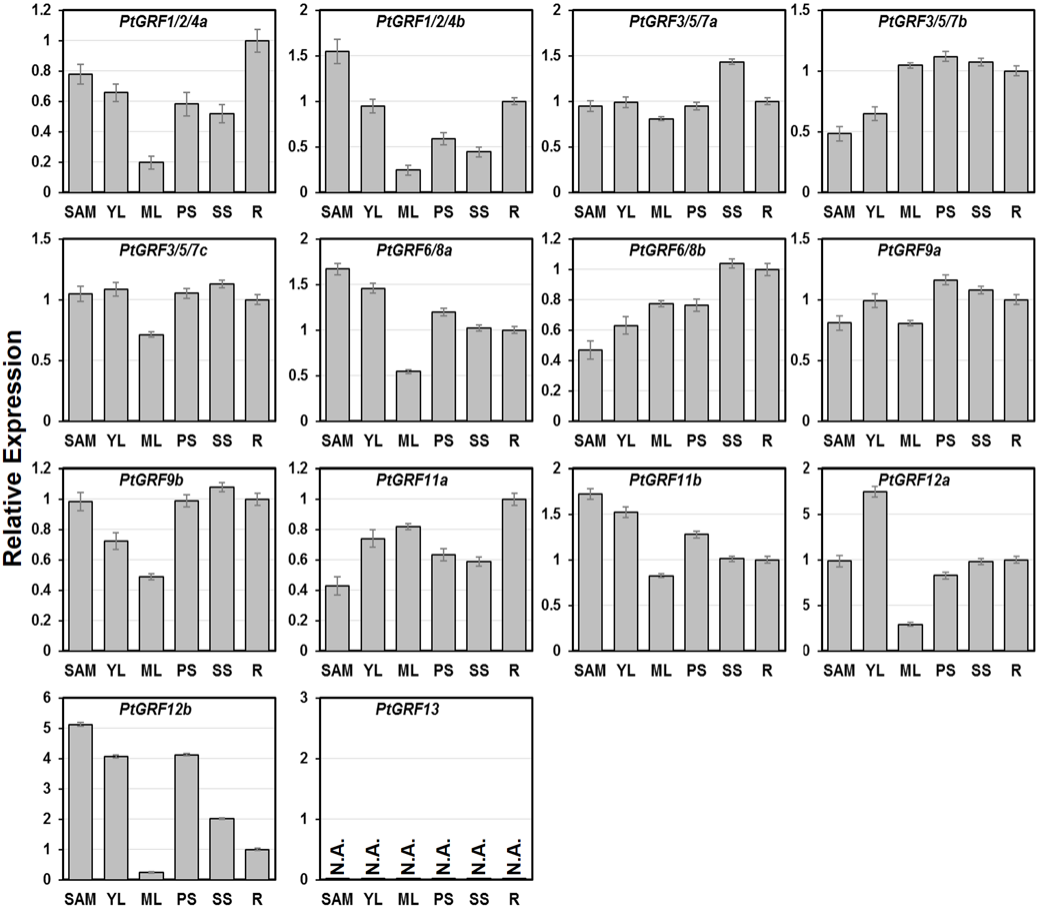
Expression profiles of *Populus 14-3-3* genes in different tissues using qRT-PCR. The relative mRNA abundance of *Populus 14-3-3* genes were quantified in six vegetative tissues (SAM-shoot apical meristem, YL-young leaves, ML-mature leaves, PS-primary stem, SS-secondary stem, R-root). The average expression of each gene was calculated relatively to the first biological replicate of roots ± standard error (SE) (n≥3). Relative expression represents log2 expression values. All primer sequences are listed in S3 Table.

To verify the expression patterns of *Populus 14-3-3s* obtained by the microarray analysis, qRT-PCR analysis was performed on six different vegetative tissues for fourteen *PtGRFs* (Fig 9). Sequence specific primers were used to distinguish the amplicons of the paralogous pairs. Noticeably, *PtGRF13* was relatively high expressed in male and female catkins by microarray analysis (Fig 8a), but it was not be detected by in silico EST analysis including male and female catkins (Fig 7). Our result also indicated that *PtGRF13* cannot be detected in the six selected vegetative tissues by qRT-PCR (Fig 9). The inconsistency is thought to be due to the very low abundance of *PtGRF13* and the old version of *Populus* genome were used in EST analysis. Taken together, the qRT-PCR results were in good agreement with the microarray data sets in this study, although the species (*P*. *alba* × *P*. *glandulosa*) used for qRT-PCR were different from the ones (*P*. *balsamifera*, *P*. *trichocarpa*, and *P*. *maximowiczii × nigra*) producing microarray data (see Materials and Methods). For example, *PtGRF1/2/4a* and *PtGRF6/8a* had low abundance in ML (Fig 9 and Fig 8a), while *PtGRF12b* and *PtGRF9b* were highly expressed in PS and SS respectively (Fig 9 and Fig 8b). The similar expression patterns between the qRT-PCR and microarray data suggested the conserved functions within the four species.

To further investigate the response of *Populus 14-3-3s* to abiotic stresses, we examined their expression patterns under low nitrogen limitation, methyl jasmonate (MeJ) treatment, mechanical wounding, hypoxia, and heat (Fig 8d and 8e). *PtGRF11a* was up-regulated both under nitrogen deprivation stress in eight-week-old mature leaves of genotype 1979 and at 90h after mechanical wounding in mature leaves of genotype RM5. In response to MeJ treatment, three genes (*PtGRF11b*, *12a*, and *12b*) were shown to be up-regulated (Fig 8d). In response to hypoxia, *PtGRF1/2/4a*, *12b*, and *13* were significantly up-regulated at 168h after hypoxia in leaves. Under heat stress, only *PtGRF11a* was up-regulated when the photosynthesis was inhibited by 20% and 30% (Fig 8e). *14-3-3s* are induced by various stresses have been identified in many plant species, indicating that they may play critical roles in plant adaptation to these stresses [1,23]. One mechanism by 14-3-3s could act in the regulation of such environmental stress responses is through the regulation of ion channels. In sugar beet cells exposed to cold or osmotic stress, 14-3-3 proteins increase at the plasma membrane and are associated with increased activity the H^+^-ATPase [1]. 14-3-3s may also exert their effects by interacting with components of hormone signaling pathways. A major pathway activated by stresses such as drought, temperature, and salt stress is the abscisic acid (ABA) signaling pathway [71]. 14-3-3s appear to be involved in the ABA signal pathway by interaction with the AREB/ABF/ABI5-like transcription factors that bind to ABA-response elements [25]. While the exact biochemical roles of *Populus 14-3-3s* in developmental processes and stress responses need to be further study.

## Conclusion

In this present study, we carried out a detailed survey of the *14-3-3* gene family in *Populus* and characterized them on the bases of phylogenetic relationship, gene structures, gene duplication, and expression profiles across different tissues and abiotic stresses. A total of fourteen *14-3-3* genes were identified in the *Populus* genome, all of which are clustered into two distinct groups (*ε* group and non-*ε* group). Exon/intron structure analyses indicated that the gene structures are relatively conserved in each subgroup. The *Populus* genome contains six paralogous *14-3-3* gene pairs, and all of them are located in conserved positions in duplicated blocks, suggesting that the segmental duplication play the key role during evolution. Moreover, comparative expression pattern analysis of *Populus 14-3-3s* revealed that *14-3-3s* might play various key roles in different development processes and various abiotic stresses in plants. Although the precise functions of *Populus 14-3-3s* remain largely unknown, our phylogenetic and expression analyses establishes a solid foundation for future comprehensive function analyses of *Populus 14-3-3s*.

## Supporting Information

S1 Fig Structural conformation of *Populus* 14-3-3 proteins. Structural comparison of superimposition of fourteen *Populus* 14-3-3 proteins (yellow) and *Nicotiana tabacum* 14-3-3 protein (blue, PDB:2o98B). 2D structural elements comparison, show a small deviations (RMSD) between protein conformations (Table 3).
(TIF)

S2 Fig Motif and Phylogenetic trees of separate nine α-helices domains of *Populus* 14-3-3 proteins. The nine α-helices motifs were illustrated using WEBLOGO (http://weblogo.berkeley.edu/logo.cgi). The phylogenetic trees were constructed by the neighbor-joining method with 1,000 bootstrap replicates based on the separate nine α-helices domains of *Populus* 14-3-3 proteins. Numbers at each branch indicate bootstrap values. Scale bar corresponds to the estimated number of amino acid substitutions per site.
(TIF)

S3 Fig Expression patterns of *Arabidopsis 14-3-3* genes in various tissues. *AtGRF6* and *AtGRF8* had low abundance in floral organs. *AtGRF12* was highly expressed in flowers and flora organs. The microarray data was obtained from AtGenExpress Visualization Tool (AVT, http://jsp.weigelworld.org/expviz/).
(TIF)

S1 Table List of *14-3-3* gene and their coding amino acid sequences identified from *P*. *trichocarpa* and other eight species (*A*. *thaliana*, *O*. *sativa*, *B*. *distachyon*, *G*. *max*, *V*. *vinifera*, *M*. *truncatula*, *S*. *bicolor*, and *P*. *patens*). (XLSX)

S2 Table Probe sets corresponding to *Populus 14-3-3* genes. (XLSX)

S3 Table The sequences of qRT-PCR primers. (XLSX)
