# Supplementary figures and images for: Genome-Wide Identification, Classification, and Expression Analysis of 14-3-3 Gene Family in Populus
Source: PLoS One. 2015 Apr 13;10(4):e0123225. doi: 10.1371/journal.pone.0123225 (PMC4395111; doi:10.1371/journal.pone.0123225)

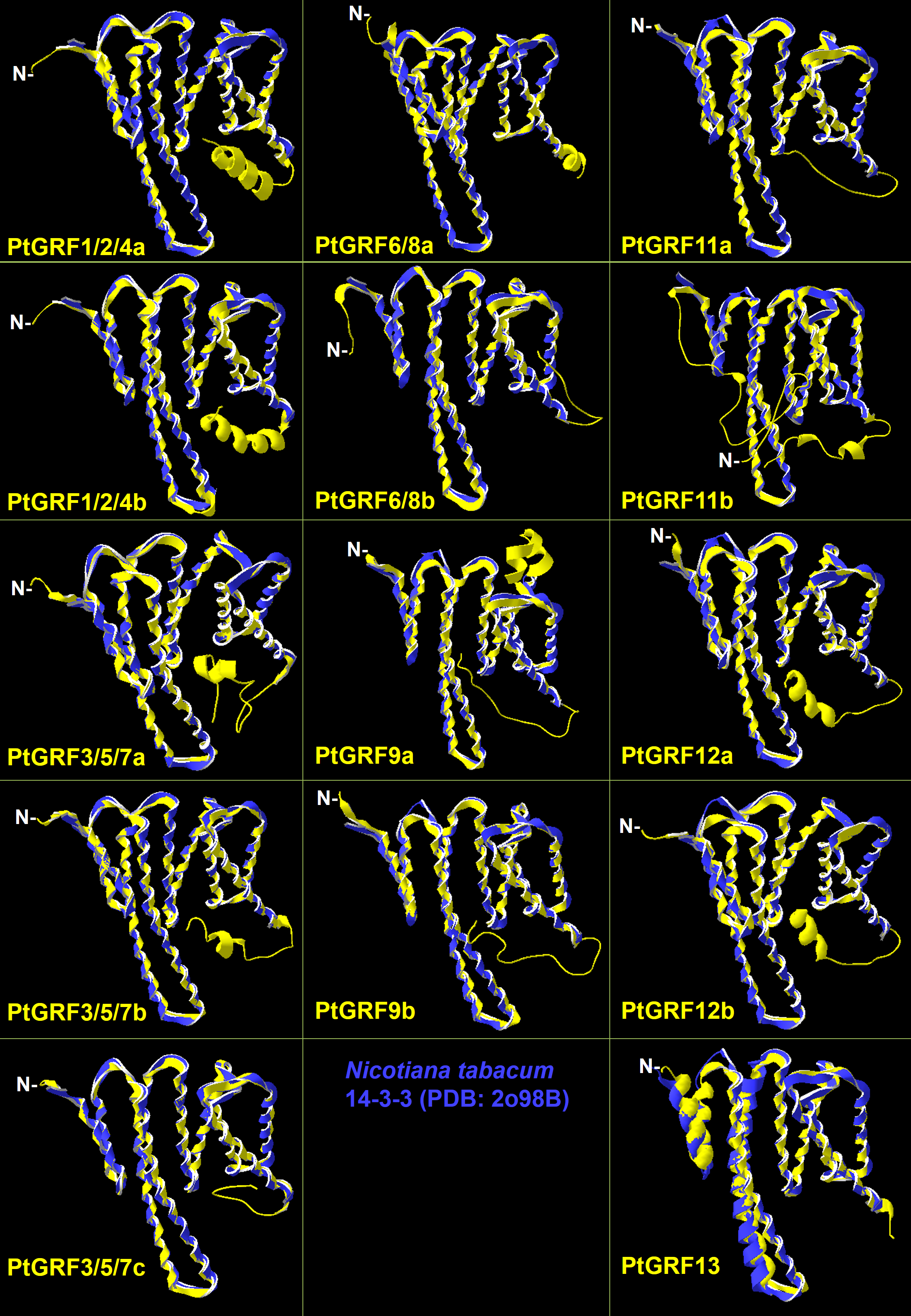

Supplement: S1 Fig — Structural comparison of superimposition of fourteen Populus 14-3-3 proteins (yellow) and Nicotiana tabacum 14-3-3 protein (blue, PDB:2o98B). 2D structural elements comparison, show a small deviations (RMSD) between protein conformations (Table 3). (TIF) [file pone.0123225.s001.TIF]

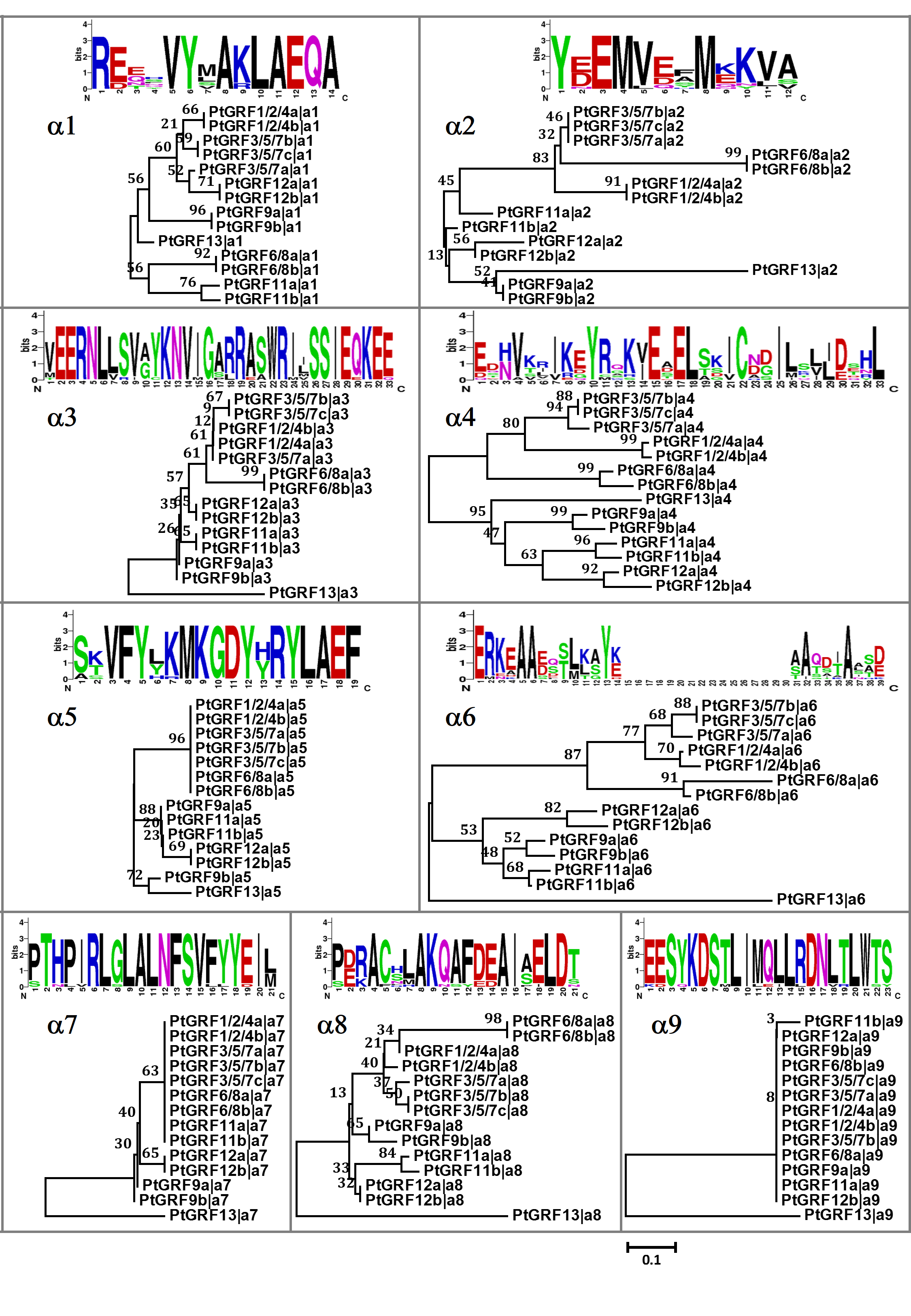

Supplement: S2 Fig — The nine α-helices motifs were illustrated using WEBLOGO (http://weblogo.berkeley.edu/logo.cgi). The phylogenetic trees were constructed by the neighbor-joining method with 1,000 bootstrap replicates based on the separate nine α-helices domains of Populus 14-3-3 proteins. Numbers at each branch indicate bootstrap values. Scale bar corresponds to the estimated number of amino acid substitutions per site. (TIF) [file pone.0123225.s002.TIF]

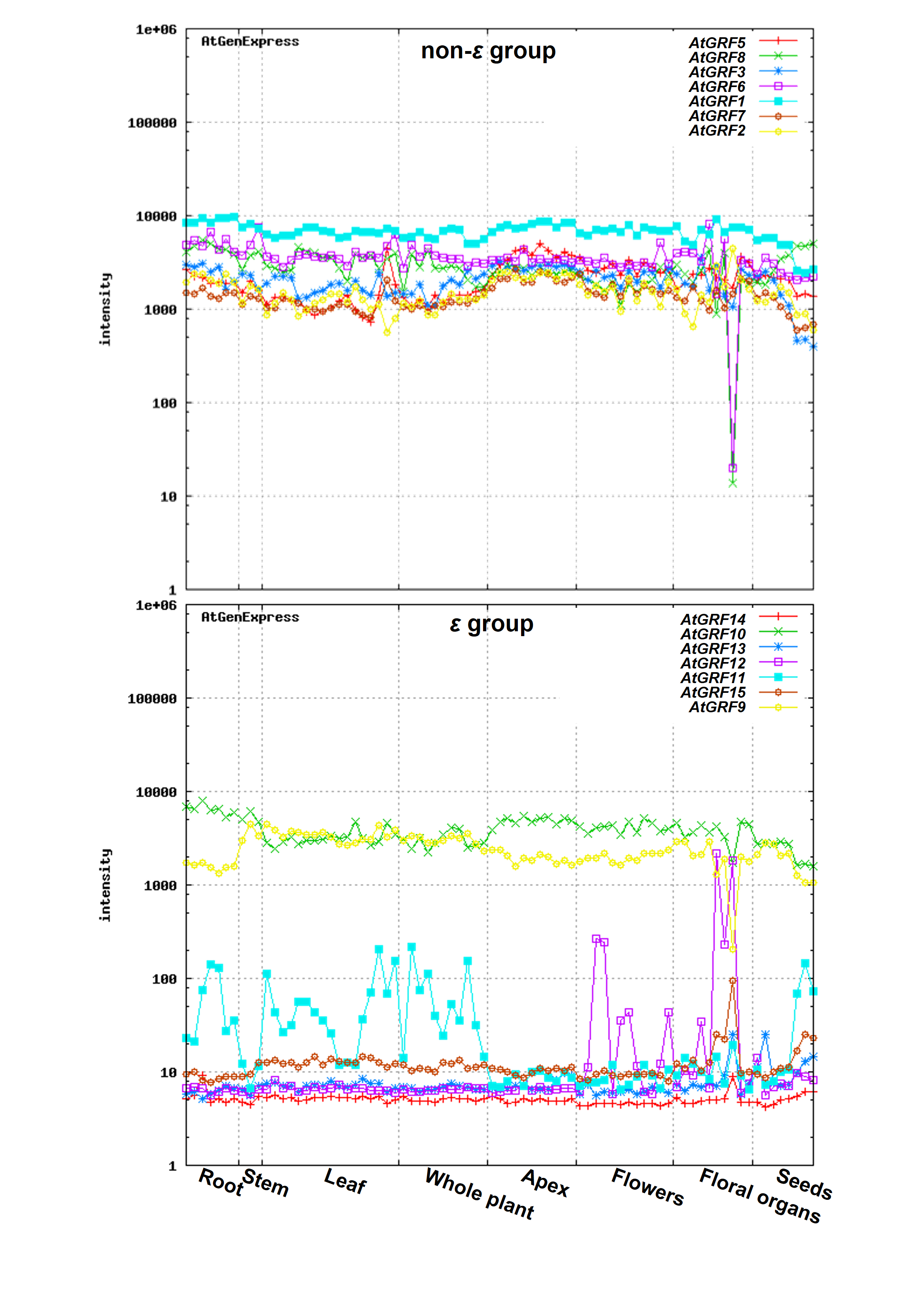

Supplement: S3 Fig — AtGRF6 and AtGRF8 had low abundance in floral organs. AtGRF12 was highly expressed in flowers and flora organs. The microarray data was obtained from AtGenExpress Visualization Tool (AVT, http://jsp.weigelworld.org/expviz/). (TIF) [file pone.0123225.s003.TIF]
